# Two new Truncatelloidea species from Melissotrypa Cave in Greece (Caenogastropoda)

**DOI:** 10.3897/zookeys.530.6137

**Published:** 2015-10-28

**Authors:** Andrzej Falniowski, Serban Sarbu

**Affiliations:** 1 Department of Malacology, Institute of Zoology, Jagiellonian University, Gronostajowa 9, 30-387 Cracow, Poland; 2 Grupul de Explorari Subacvatice si Speologice, str Frumoasa 31-B, Bucuresti, Romania

**Keywords:** Gastropoda, Hydrobiidae, Moitessieriidae, aquatic snails, morphology, cytochrome oxidase, taxonomy, troglobionts

## Abstract

In the small lake located in the cave Melissotrypa in Thessalia, Greece, truncatelloidean gastropods representing two species were found, new to science. One of them, represented by two specimens only, has been described based on the shell characters only; with its cytochrome oxidase sequence it has been assigned to the genus *Iglica*, and to the family Moitessieriidae, *Iglica hellenica* **sp. n.** For the other species, represented by 30 collected specimens, the shell, protoconch, radula, head, penis and female reproductive organs have been described; all the morphological characters and cytochrome oxidase sequences have confirmed its assignment to the genus *Daphniola* (Hydrobiidae: Sadlerianinae), *Daphniola magdalenae* Falniowski, **sp. n.**

## Introduction

In June 2014, in Melissotrypa Cave in Greece (39°52'38"N and 22°02'58"E), several specimens of Truncatelloidea gastropods were collected. This was the third visit by the second author to this cave, but the snails were found for the first time.

The cave is located in Melissotrypa Kefalovriso Elassona, north of Larissa, and is the largest known underground karstic form of karst system Kranias Elassona, drilled in marbles. The character of the cave is demonstrated by the remaining forms of dissolution and growth of the cave, the gypsum and detected hydrogen sulfide in the lakes of the cave. The cave covers an area 0.06 km^2^ and has a total length of mapped passageways about 2103.6 m. The elevation in the region of the inlet orifice is 299 m while the interior reaches a depth up -47.3 m i.e. absolute altitude 251.7 m. The depth of the precipitous entry is 14.6 m (http://7gym-laris.lar.sch.gr/perivalon/spilaia.htm).

Many specimens of gastropods were concentrated in just one area in the sulfuric lake, close to the shore in a depth of approximately 10 cm. In the vicinity of the lake there are no terrestrial animals, although there are microbial biofilms and organic matter. The aquatic fauna is highly interesting: the most abundant form is an amphipod *Niphargus*, which swims upside down, seemingly an adaptation to such water chemistry. The snails do not live everywhere, but only in one place on a limestone wall, at 5–10 cm beneath the water surface. There were hundreds of individuals gathered in a compact group. Maybe there are more such groups, but the water is deep and one cannot reach the walls except by means of a small boat, the lake being very narrow. In this cave, there is also another lake, at several hundred meters away from the former, in which the water has no sulfur, and which is sometimes dry. No snails have been found in it.

Only two specimens with a turriform shell were collected, and approximately 30 specimens with a valvatiform shell. The aim of the paper is to describe these two snails collected in Melissotrypa Cave.

## Materials and methods

The snails were collected by hand and placed directly in 95% ethanol. The ethanol was changed twice, and the material stored at -20 °C.

The shells were photographed with a CANON EOS 50D digital camera, attached to a NIKON SMZ18 stereoscope microscope with dark field. They were dissected using a NIKON SMZ18 stereoscope microscope with a NIKON drawing apparatus, and a NIKON DS-5 digital camera. Radulae and protoconchs were examined using a JEOL JSM-5410 scanning electron microscope, applying the techniques described by Falniowski (1990).

DNA was extracted from foot tissue of two specimens. The tissue was hydrated in TE buffer (3 × 10 min); total genomic DNA was then extracted with the SHERLOCK extracting kit (A&A Biotechnology), and the final product was dissolved in 20 µl TE buffer. The PCR reaction was performed with the following primers: LCOI490 (5’-ggtcaacaaatcataaagatattgg-3’) (Folmer et al. 1994) and COR722b (5’-taaacttcagggtgaccaaaaaatya-3’) (Wilke and Davis 2000) for the cytochrome oxidase subunit I (COI) mitochondrial gene.

The PCR conditions were as follows: initial denaturation step of 4 min at 94 °C, followed by 35 cycles of 1 min at 94 °C, 1 min at 55 °C 2 min at 72 °C, and a final extension of 4 min at 72 °C. The total volume of each PCR reaction mixture was 50 µl. To check the quality of the PCR products 10 µl of the PCR product was ran on 1% agarose gel. The PCR products were purified using Clean-Up columns (A&A Biotechnology) and were then amplified in both directions using BigDye Terminator v3.1 (Applied Biosystems), following the manufacturer’s protocol and with the primers described above. The sequencing reaction products were purified using ExTerminator Columns (A&A Biotechnology); DNA sequences then underwent electrophoresis on an ABI Prism sequencer.

The COI sequences were aligned by eye using BioEdit 5.0.0 (Hall 1999). The saturation test of Xia et al. (2003) was performed using DAMBE (Xia 2013). Sequences obtained from the snails from Melissotrypa Cave in the present work were used in a phylogenetic analysis with other sequences obtained from GenBank (Table 1). A maximum likelihood (ML) approach was conducted in RAxML v8.0.24 (Stamatakis 2014). One thousand searches were initiated with starting trees obtained through randomized stepwise addition maximum parsimony method. The tree with the highest likelihood score was considered as the best representation of the phylogeny. Bootstrap support was calculated with 1000 replicates and summarized onto the best ML tree. RAxML analyses were performed using free computational resource CIPRES Science Gateway (Miller et al. 2010). Genetic *p*-distances between the species of *Daphniola* were calculated using MEGA6 (Tamura et al. 2013), with standard errors estimated by 1,000 bootstrap replications with pairwise deletion of missing data. The maximum composite likelihood distance and Tajima relative rate tests of local clock-like behavior (Tajima 1993) were performed using MEGA6.

**Table 1.** Taxa used for phylogenetic analyses, with their GenBank Accession Numbers and references.

Species	COI GB#	References
*Adrioinsulana conovula* (Frauenfeld, 1863)	AF367628	Wilke et al. (2001)
*Agrafia wiktori* Szarowska & Falniowski, 2011	JF906762	Szarowska and Falniowski (2011)
*Alzoniella finalina* Giusti & Bodon, 1984	AF367650	Wilke et al. (2001)
*Anagastina zetavalis* (Radoman, 1973)	EF070616	Szarowska (2006)
*Avenionia brevis* (Draparnaud, 1805)	AF367638	Wilke et al. (2001)
*Belgrandiella kusceri* (Wagner, 1914)	KT218520	Falniowski and Beran (2015)
*Bithynia tentaculata* (Linnaeus, 1758)	AF367643	Wilke et al. (2001)
*Boleana umbilicata* (Kuščer, 1932)	KT218521	Falniowski and Beran (2015)
*Bythinella austriaca* (Frauenfeld, 1857)	FJ545132	Falniowski et al. (2009)
*Bythiospeum* sp.	AF367634	Wilke et al. (2001)
*Bythiospeum acutum* (Geyer, 1904)	HM107120	unpublished, from GenBank
*Bythiospeum francomontanum* Bernasconi, 19730	HM107131	unpublished, from GenBank
*Bythiospeum hungaricum* (Soós, 1927)	KP296923	unpublished, from GenBank
*Bythiospeum husmanni* (C.R. Boettger, 1963)	HM107134	unpublished, from GenBank
*Bythiospeum pellucidum* (v. Wiedersheim, 1973)	HM107124	unpublished, from GenBank
*Bythiospeum suevicum* (Geyer, 1905)	HM107118	unpublished, from GenBank
*Dalmatinella fluviatilis* Radoman, 1973	KC344541	Falniowski and Szarowska (2013)
*Daphniola exigua* (A. Schmidt, 1856)	EU047767	Falniowski et al. (2007)
*Daphniola hadei* (Gittenberger, 1982)	JF916477	Falniowski and Szarowska (2011a)
*Daphniola graeca* Radoman, 1973	EF070618	Szarowska (2006)
*Daphniola louisi* Falniowski & Szarowska, 2000	EU047769	Falniowski et al. (2007)
*Daphniola* sp.	KM887915	Szarowska et al. (2014)
*Daphniola magdalenae* sp. n.	KT825578-80	present study
*Dianella thiesseana* (Kobelt, 1878)	AY676127	Szarowska et al. (2005)
*Fissuria boui* Boeters, 1981	AF367654	Wilke et al. (2001)
*Graecoarganiella parnassiana* Falniowski & Szarowska, 2011	JN202348	Falniowski and Szarowska (2011b)
*Graziana alpestris* (Frauenfeld, 1863)	AF367641	Wilke et al. (2001)
*Grossuana codreanui* (Grossu, 1946)	EF061919	Szarowska et al. (2007)
*Hauffenia tellinii* (Pollonera, 1898)	AF367640	Wilke et al. (2001)
*Heleobia dalmatica* (Radoman, 1974) *Horatia klecakiana* Bourguignat, 1887	AF367631 KJ159128	Wilke et al. (2001) Szarowska and Falniowski (2014)
*Hydrobia acuta* (Draparnaud, 1805)	AF278808	Wilke and Davis (2000)
*Iglica hellenica* sp. n.	KT825581	present study
*Islamia piristoma* Bodon & Cianfanelli, 2001	AF367639	Wilke et al. (2001)
*Lithoglyphus naticoides* (C. Pfeiffer, 1828)	AF367642	Wilke et al. (2001)
*Marstoniopsis insubrica* (Küster, 1853)	AY027813	Falniowski and Wilke (2001)
Moitessieria cf. puteana (Coutagne, 1883)	AF367635	Wilke et al. (2001)
*Montenegrospeum bogici* (Pešić & Glöer, 2012)	KM875510	Falniowski et al. (2014)
*Pseudamnicola lucensis* (Issel, 1866)	AF367651	Wilke et al. (2001)
*Pyrgula annulata* (Linnaeus, 1767)	AY341258	Szarowska et al. (2005)
*Radomaniola callosa* (Paulucci, 1881)	AF367649	Wilke et al. (2001)
*Rissoa labiosa* (Montagu, 1803)	AY676128	Szarowska et al. (2005)
*Sadleriana fluminensis* (Küster, 1853)	AY273996	Wilke et al. (2001)
*Tanousia zrmanjae* (Brusina, 1866)	Xx	Beran et al. (2015)
*Trichonia kephalovrissonia* Radoman, 1973	EF070619	Szarowska (2006)
*Ventrosia ventrosa* (Montagu, 1803)	AF118335	Wilke and Davis (2000)

## Systematic part

### Family Moitessieriidae Bourguignat, 1863 Genus *Iglica* Wagner, 1927

#### Iglica hellenica sp. n.

Taxon classificationAnimaliaLittorinimorphaHydrobiidae

http://zoobank.org/44EEDD4D-448D-4ABB-9128-E6AFC35F5B51

##### Holotype.

Ethanol-fixed specimen, Melissotrypa Cave, Thessalia, Greece, 39°52'38"N, 22°02'58"E, sulphidic lake, near the shore, June 2014, S. Sarbu coll., ZMUJ-M.2107.

##### Paratype.

One specimen destroyed for DNA extraction details as for holotype.

##### Diagnosis.

Shell relatively big, turriform, readily distinguished from geographically close and related species *Iglica sidariensis*, *Iglica maasseni*, *Iglica wolfischeri* and *Iglica alpheus* by its larger size and more convex whorls *Iglica hellenica* is readily distinguished from the geographically closest species *Paladilhiopsis thessalica* by its larger size and narrow aperture.

##### Description.

**Figure 1.**
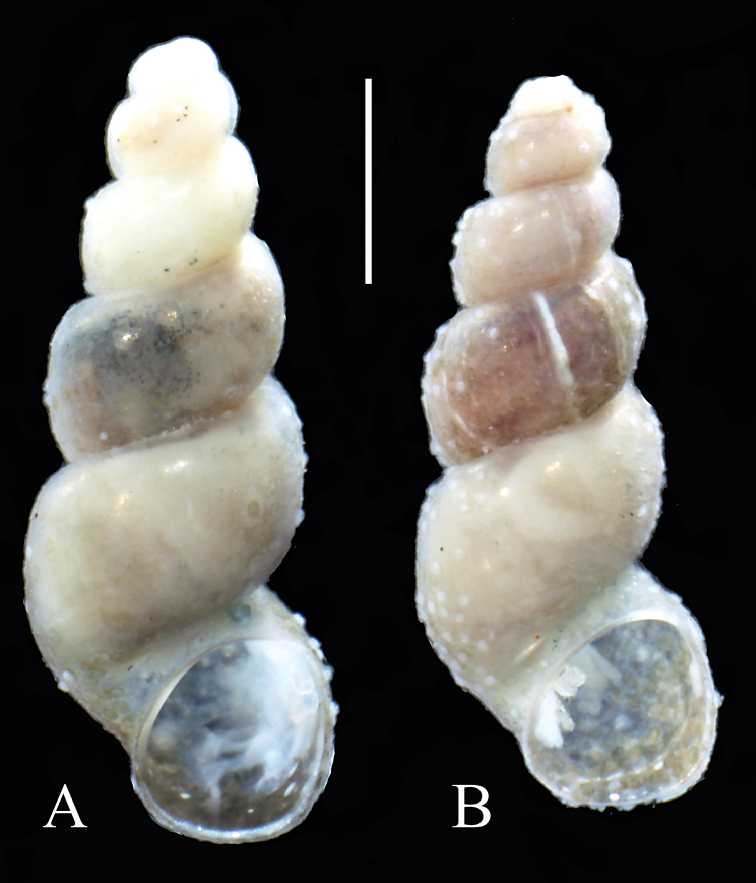
Shells of *Iglica hellenica* sp. n.: **A** holotype **B** sequenced specimen. Scale bar 1 mm.

Shell (Fig. 1) up to 4.04 mm tall, 5.5 whorls, spire height 281% width of shell. Holotype measurements: shell height 4.04 mm, spire height 1.85 mm, body whorl breadth 1.44 mm, aperture height 1.22 mm, aperture breadth 1.05 mm, whorls number 5½. Teleoconch whorls highly convex, evenly rounded. Aperture narrow, ovate, weakly angled adapically, separated from body whorl by a broad groove. Parietal lip complete, adnate, no umbilicus. Outer lip simple, orthocline. Shell glossy with no sculpture, periostracum yellowish. Soft parts pinkish, with no pigment. External morphology and anatomy unknown.

##### Etymology.

The specific epithet (*hellenica*) is a Greek adjective meaning Greek.

##### Distribution and habitat.

Known from two specimens from the type locality only.

### Family Hydrobiidae Troschel, 1857 Subfamily Sadlerianinae Radoman, 1973 Genus *Daphniola* Radoman, 1973

#### Daphniola magdalenae

Taxon classificationAnimaliaLittorinimorphaHydrobiidae

Falniowski sp. n.

http://zoobank.org/AF91ADE8-10B4-4737-8022-7EFDDC316EAD

##### Types.

Ethanol-fixed specimens, Melissotrypa Cave, Thessalia, Greece, 39°52'38"N, 22°02'58"E, sulphidic lake, near the shore, June 2014, S. Sarbu coll., holotype: ZMUJ-M.2109; 20 paratypes: ZMUJ-M.2110-ZMUJ-M.2130.

##### Diagnosis.

Shell relatively big, valvatiform-trochiform; soft parts with no pigment, no eyes, penis with long and slender filament and big outgrowth on the left side. Readily distinguished from geographically and closely related *Daphniola exigua* (= *Daphniola graeca*) by its bigger size (2.5 *vs.* 1.5 mm), reddish operculum, broader base and longer and thinner filament of the penis. Differentiated from *Daphniola louisi* (from Kessariani at Athens) by its larger size, higher spire, longer and thinner filament and more prominent outgrowth on the left side of the penis. Differs from *Daphniola hadei* (from Gythion at Peloponnese) by its double size, higher spire and much more prominent outgrowth on the left side of the penis.

##### Description.

**Figure 2.**
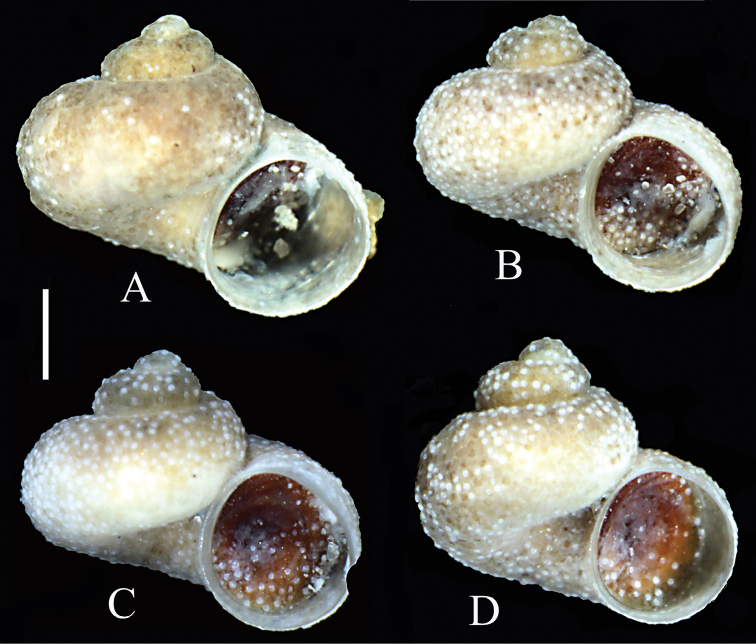
Shells of *Daphniola magdalenae* sp. n: **A** holotype **B–D** paratypes. Scale bar: 0.5 mm

Shell (Fig. 2A–D) valvatiform-trochiform, up to 2.68 mm tall, having 3.5–3.75 whorls, spire height 16% height of shell, and 13–16% width of shell. Teleoconch whorls moderately convex, evenly rounded, growing rapidly in diameter. Aperture circular, parietal lip complete, umbilicus very broad, outer lip simple, orthocline. Teleoconch with delicate growth lines, periostracum pinkish. Shell parameters for a series of paratypes are given in Table 2. On the surface there are numerous pellets of sediment, most probably of sulfuric bacteria.

**Table 2.** Shell measurements of *Daphniola magdalenae*, n = 10.

shell heigth	shell heigth (mm)	spire heigth (mm)	body whorl width (mm)	aperture heigth (mm)	aperture width (mm)	whorl number
holotype	2.51	0.38	1.99	1.37	1.34	3.5
mean	2.335	0.363	1.895	1.346	1.280	3.70
sd	0.1788	0.0503	0.1506	0.0797	0.0643	0.1083
minimum	2.16	0.28	1.76	1.20	1.19	3.50
maximum	2.68	0.43	2.21	1.44	1.39	3.75

**Figures 3–7.**
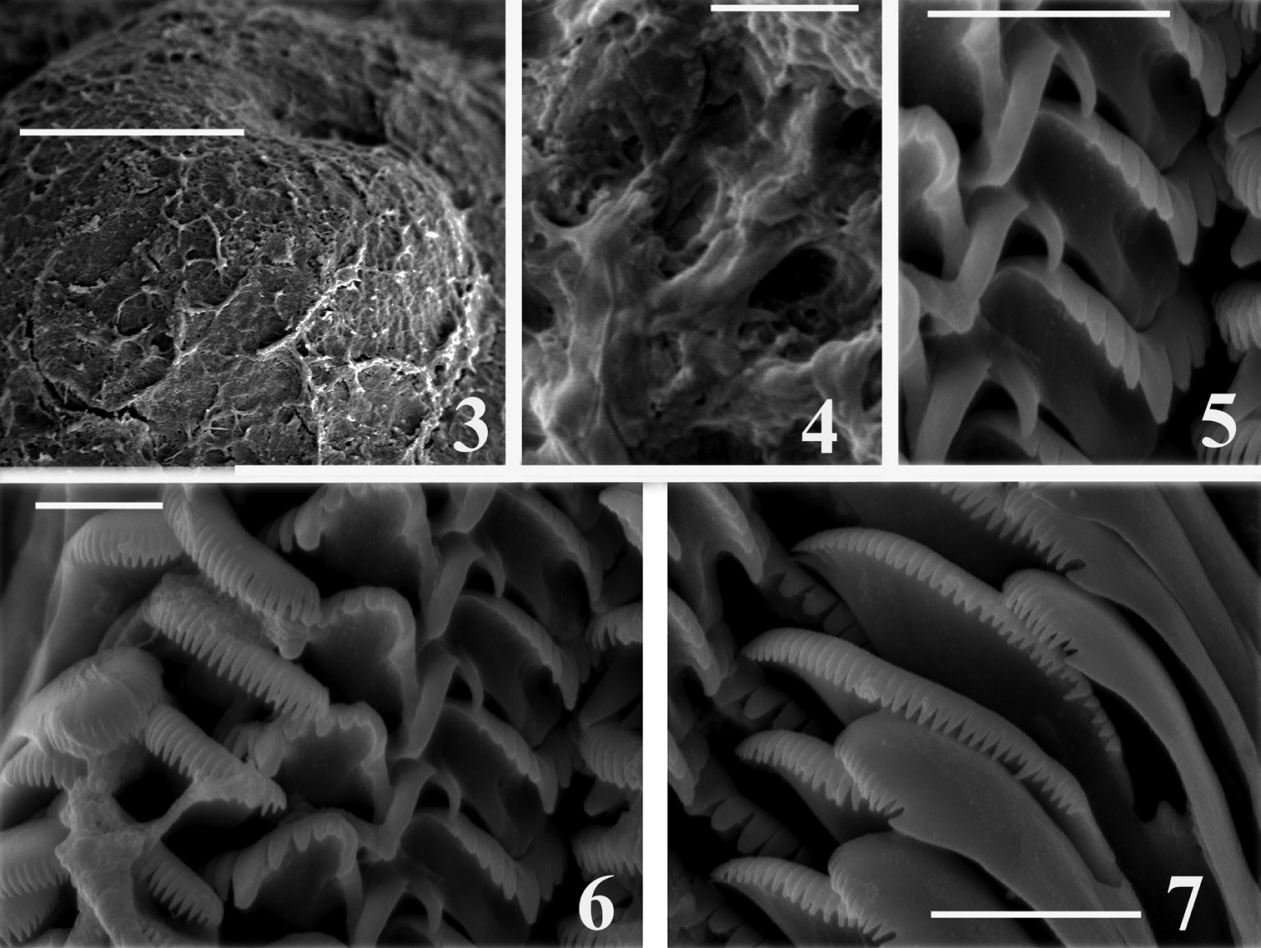
Protoconch and radula of *Daphniola magdalenae*: **3–4** protoconch **5**–7 radula **5** central teeth **6** central, lateral and marginal teeth **7** marginal teeth. Scale bar: 100 µm (**3**); 3 µm (**4, 5**); 4 µm (**6, 7**).

Inner and outer sides of operculum smooth. Operculum pink (Fig. 2A–D). Protoconch of 1.25–1.40 whorls growing slowly (Fig. 3), with a net-like pattern of dense depressions, their shape irregular (Fig. 4), covering all the protoconch and initial part of the teleoconch.

Radula (Figs 5–7): taenioglossate, typically hydrobiid; the cusps on the central, lateral and inner marginal teeth prominent, long and sharp; the central tooth trapezoid (Figs 5–6), with one pair of big basal cusps arising from the tooth face (Fig. 5) and numerous long cusps along the cutting edge, the basal tongue broadly V-shaped and about equal in length to the lateral margins, lateral cusps five–six. Lateral teeth (Figs 6–7) having four cusps on inner, and five cusps on outer side, central cusp broad and blunt. Inner marginal tooth (Fig. 7) with 35–36 cusps, outer marginal teeth (Figs 6–7) with 21–23 cusps.

**Figures 8–11.**
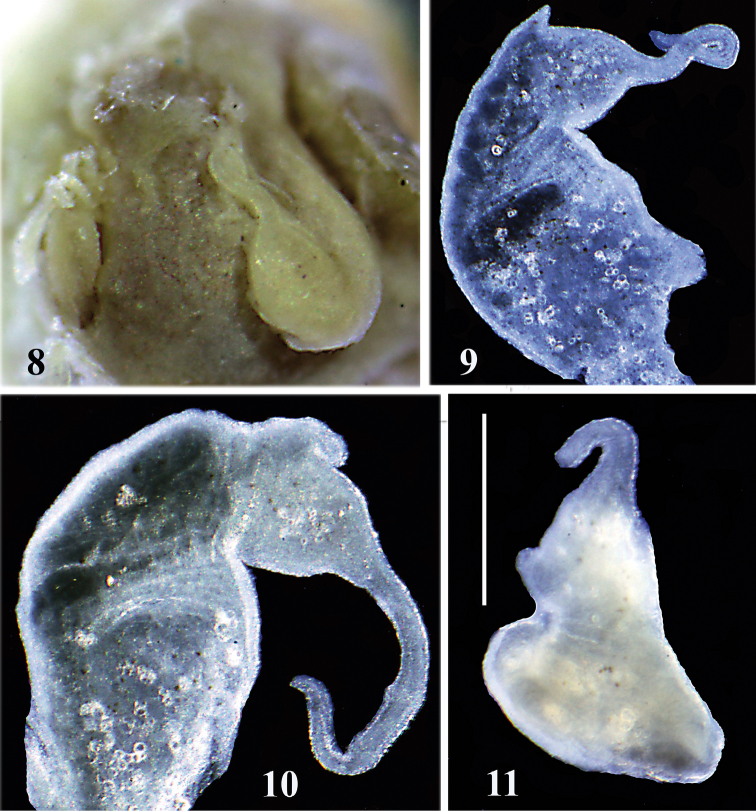
Head and penes of *Daphniola magdalenae*: **8** head with penis, **9–11** penes. Scale bar: 250 µm.

**Figure 12.**
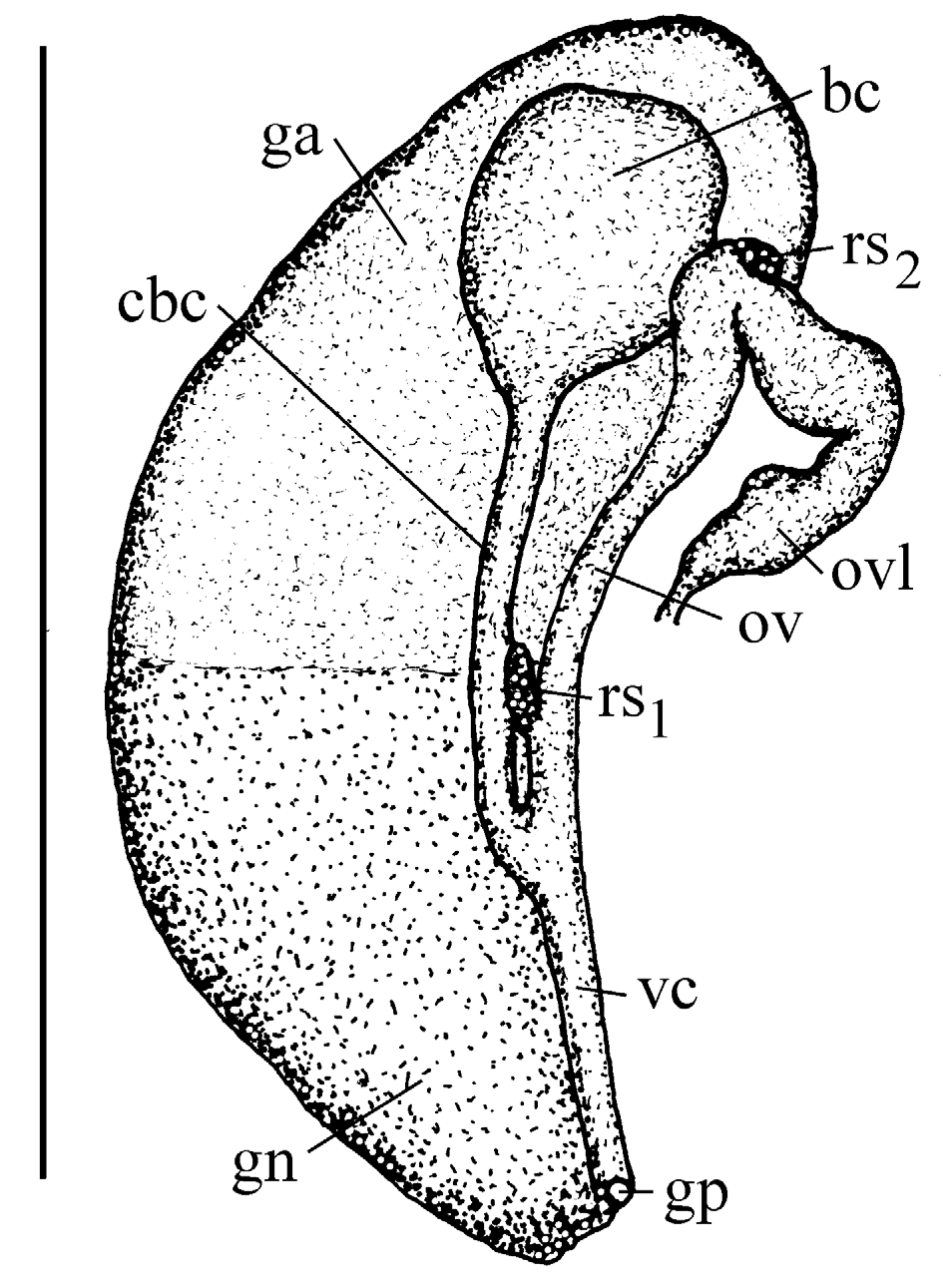
Renal and pallial section of female reproductive organs of *Daphniola magdalenae* (bc – bursa copulatrix, cbc – duct of bursa copulatrix, ga – albuminoid gland, gn – nidamental gland, gp – gonoporus, ov – oviduct, ovl – loop of (renal) oviduct, rs1, rs2 – receptacula seminis, nomenclature after Radoman (1973, 1983), vc – ventral canal). Scale bar 1 mm.

Animal brownish, with no pigment, and no eyes (Fig. 8). Penis (Figs 9–11) having broad base bent U-shaped in natural position (Fig. 8), long and narrow filament and prominent outgrowth on its left edge. Female reproductive organs (Fig. 12) with big bursa copulatrix with long duct and two small receptacula seminis.

##### Etymology.

Named in memory of Dr Magdalena Szarowska, a malacologist, wife and best friend of the first author.

##### Distribution and habitat.

Known from the type locality only.

### Molecular relationships of the new taxa

**Figure 13.**
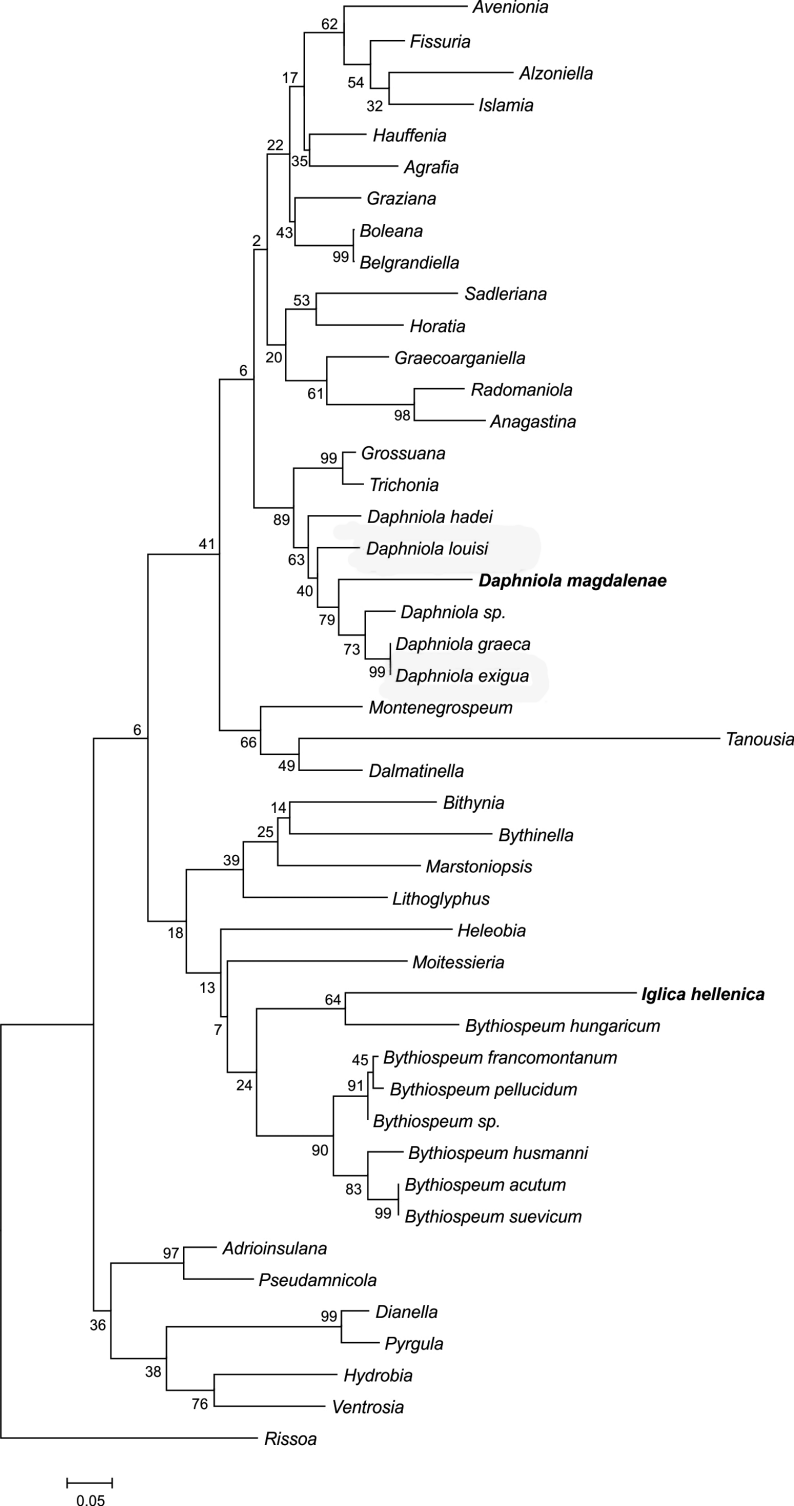
Maximum likelihood tree computed for cytochrome oxidase I sequences, bootstrap supports given if > 50%.

The saturation test of Xia et al. (2003) revealed a significant degree of saturation in the third position of the sequences. In rissooids, COI approaches saturation with approximately 18.6% or 120 nucleotide differences (Davis et al. 1998), which seems to happen after approximately 10 million years. However, to avoid a substantial loss of information in the case of closely related species, this position was not excluded from the dataset and it was used for the analysis. The maximum likelihood tree (Fig. 13) was characterized by low bootstrap values at deep nodes, which is typical of cytochrome oxidase-based phylogenies, but clearly showed that *Daphniola magdalenae* sp. n. belonged to the genus *Daphniola* (bootstrap value 63%), although it was clearly a distinct species. Its closest relatives were *Daphniola* sp. from Khios and Rhodes islands, and *Daphniola exigua*/*Daphniola graeca* from Tembi valley (bootstrap support 79%). The bootstrap support of the clade of *Daphniola*, *Trichonia* Radoman, 1973, and *Grossuana* Radoman, 1973 was 89%. The p-distance between *Daphniola magdalenae* sp. n. and *Daphniola exigua* was p = 0.1325. The relative rates test for all the *Daphniola* species confirmed the ultrametricity of the data. The tree also confirmed close relationships of *Iglica hellenica* sp. n. with “Bythiospeum” hungaricum (bootstrap value/support 64%), and that both *Iglica hellenica* and “Bythiospeum” hungaricum do not belong to the genus *Byhiospeum* Bourguignat, 1882.

## Discussion

With one (since the other had to be destroyed for DNA extraction) available specimen of *Iglica hellenica* sp. n. it has not been possible to study its soft parts. However, nearly all the representatives of *Bythiospeum*, *Paladilhiopsis*, *Iglica*, etc. are known as empty shells only. The distinction between these genera remains unclear. The molecular tree, as well as the phylogeny presented by Wilke et al. (2013), does not confirm even the close relationships between *Bythiospeum*, *Iglica hellenica* sp. n., and *Moitessieria*. It also does not confirm that “Bythiospeum” hungaricum belongs to the genus *Bythiospeum*, but confirms its close relationships with *Iglica hellenica*. From Greece there are four known species of *Iglica*: *Iglica sidarensis* Schütt, 1980 from Corfu, *Iglica maasseni* Schütt, 1980 from Rhodes, and two species from the Peloponnese: *Iglica wolfischeri* A. & P. Reischutz, 2004 and *Iglica alpheus* A. & P. Reischutz, 2004. With the exception of *Iglica alpheus*, the shells of all are similar to the one of *Iglica hellenica*, but much smaller with shell heights of 1.5–2.3 mm, compared with 4.04 mm in *Iglica hellenica*. The representatives of another cave-inhabiting genus *Paladilhiopsis* Pavlovic, 1913 should also be considered. From Greece there are three species in this genus: *Paladilhiopsis blanci* (Westerlund, 1886) from the islands Cephalonia and Lefkada, *Paladilhiopsis janinensis* Schütt, 1962 from the springs at the shore of Pamvotis Lake (now the springs are completely dry), and *Paladilhiopsis thessalica* Schütt, 1970, from Pyrgetos at Tembi Valley. This locality is only 46 km away from Melissotrypa Cave. However, the shell but especially the aperture of *Iglica hellenica* is typical of *Iglica*, not of *Paladilhiopsis* (e. g. Schütt 1980). Moreover, the 18S sequence of *Iglica hellenica* (unpublished data) was very different from the one of *Paladilhiopsis carpatica* Soós, 1940 from Vadu Crisul Cave in Romania (Szarowska 2006). Thus the assignment of *Iglica hellenica* to the genus *Iglica* remains justified based on the available data.

The shells of *Daphniola exigua* are highly variable (Falniowski et al. 2007), including the similar shells of *Daphniola magdalenae* sp. n., but are much smaller (maximum 1.58 mm *vs.* 2.68 in *Daphniola magdalenae*). The shells of the other species of *Daphniola* have lower spires, and are also maximum 1.5 mm tall (Falniowski et al. 2007, Falniowski and Szarowska 2011a). The penis of *Daphniola magdalenae* sp. n. differs in its long and narrow, sharply pointed filament of the penes from those of *Daphniola exigua* and *Daphniola graeca* (Radoman 1983, Szarowska 2006), and *Daphniola louisi* (Falniowski & Szarowska, 2000). A similar filament, but less prominent outgrowth on the left side of the penis is characteristic of *Daphniola hadei* (Falniowski and Szarowska 2011a). The female reproductive organs of *Daphniola magdalenae* are characteristic of *Daphniola* (Radoman 1973, 1983, Szarowska 2006). Some differences in size proportions of the receptacula and bursa could be observed between the species, but the variability is high;veven the genera of the Hydrobiidae with two receptacula could not always be recognized with this character (Falniowski et al. 2012). *Daphniola exigua* inhabits two springs in Tembi Valley, approximately 50 km from Melissotripa cave, but in the molecular tree it is not the sister species of *Daphniola magdalenae* sp. n.. The genetic distance between *Daphniola magdalenae* and *Daphniola exigua* is p = 0.1325. Based on mtCOI clock calibrations of 1.83% per million years for European Hydrobiidae (Wilke 2003) and 1.62% per million years for *Pyrgulopsis* (Hershler and Liu 2008), the estimated divergence times of the two species ranged from 7.24 to 8.20 mya, thus the very beginning of the Messinian or even upper Tortonian in the Miocene.

The molecular tree confirms relationships of both new species *Iglica hellenica* and *Daphniola magdalenae*. As it is based on one short fragment of mitochondrial DNA, it presents the phylogeny of this fragment, certainly not of the species/genera (e.g., Avise 2000), and its deep nodes are not supported. Thus the tree cannot be interpreted as phylogeny of the Truncatelloidea. However, it seems sufficient to detect the closest relatives of the new species described in this paper.

## Supplementary Material

XML Treatment for Iglica hellenica

XML Treatment for Daphniola magdalenae
